# H5Nx Viruses Emerged during the Suppression of H5N1 Virus Populations in Poultry

**DOI:** 10.1128/Spectrum.01309-21

**Published:** 2021-09-29

**Authors:** Yao-Tsun Li, Yvonne C. F. Su, Gavin J. D. Smith

**Affiliations:** a Programme in Emerging Infectious Diseases, Duke-NUS Medical School, Singapore; b SingHealth Duke-NUS Global Health Institute, SingHealth Duke-NUS Academic Medical Centre, Singapore; c Duke Global Health Institute, Duke University, Durham, North Carolina, USA; University of Georgia

**Keywords:** evolution, pandemic, zoonotic, influenza, zoonotic infections

## Abstract

Highly pathogenic avian influenza (HPAI) H5 viruses have posed a substantial pandemic threat through repeated human infection since their emergence in China in 1996. Nationwide control measures, including vaccination of poultry, were implemented in 2005, leading to a sharp reduction in H5N1 virus outbreaks. In 2008, novel non-N1 subtype (H5Nx) viruses emerged, gradually replacing the dominant H5N1 subtype and causing global outbreaks. The cause of this major shift in the ecology of HPAI H5 viruses remains unknown. Here, we show that major H5N1 virus lineages underwent population bottlenecks in 2006, followed by a recovery in virus populations between 2007 and 2009. Our analyses indicate that control measures, not competition from H5Nx viruses, were responsible for the H5N1 decline, with an H5N1 lineage capable of infecting poultry and wild birds experiencing a less severe population bottleneck due to circulation in unaffected wild birds. We show that H5Nx viruses emerged during the successful suppression of H5N1 virus populations in poultry, providing an opportunity for antigenically distinct H5Nx viruses to propagate. Avian influenza vaccination programs would benefit from universal vaccines targeting a wider diversity of influenza viruses to prevent the emergence of novel subtypes.

**IMPORTANCE** A major shift in the ecology of highly pathogenic avian influenza (HPAI) H5 viruses occurred from 2008 to 2014, when viruses with non-N1 neuraminidase genes (termed H5Nx viruses) emerged and caused global H5 virus outbreaks. Here, we demonstrate that nationwide control measures, including vaccination in China, successfully suppressed H5N1 populations in poultry, providing an opportunity for antigenically distinct H5Nx viruses to emerge. In particular, we show that the widespread use of H5N1 vaccines likely conferred a fitness advantage to H5Nx viruses due to the antigenic mismatch of the neuraminidase genes. These results indicate that avian influenza vaccination programs would benefit from universal vaccines that target a wider diversity of influenza viruses to prevent potential emergence of novel subtypes.

## INTRODUCTION

**FIG 1:**
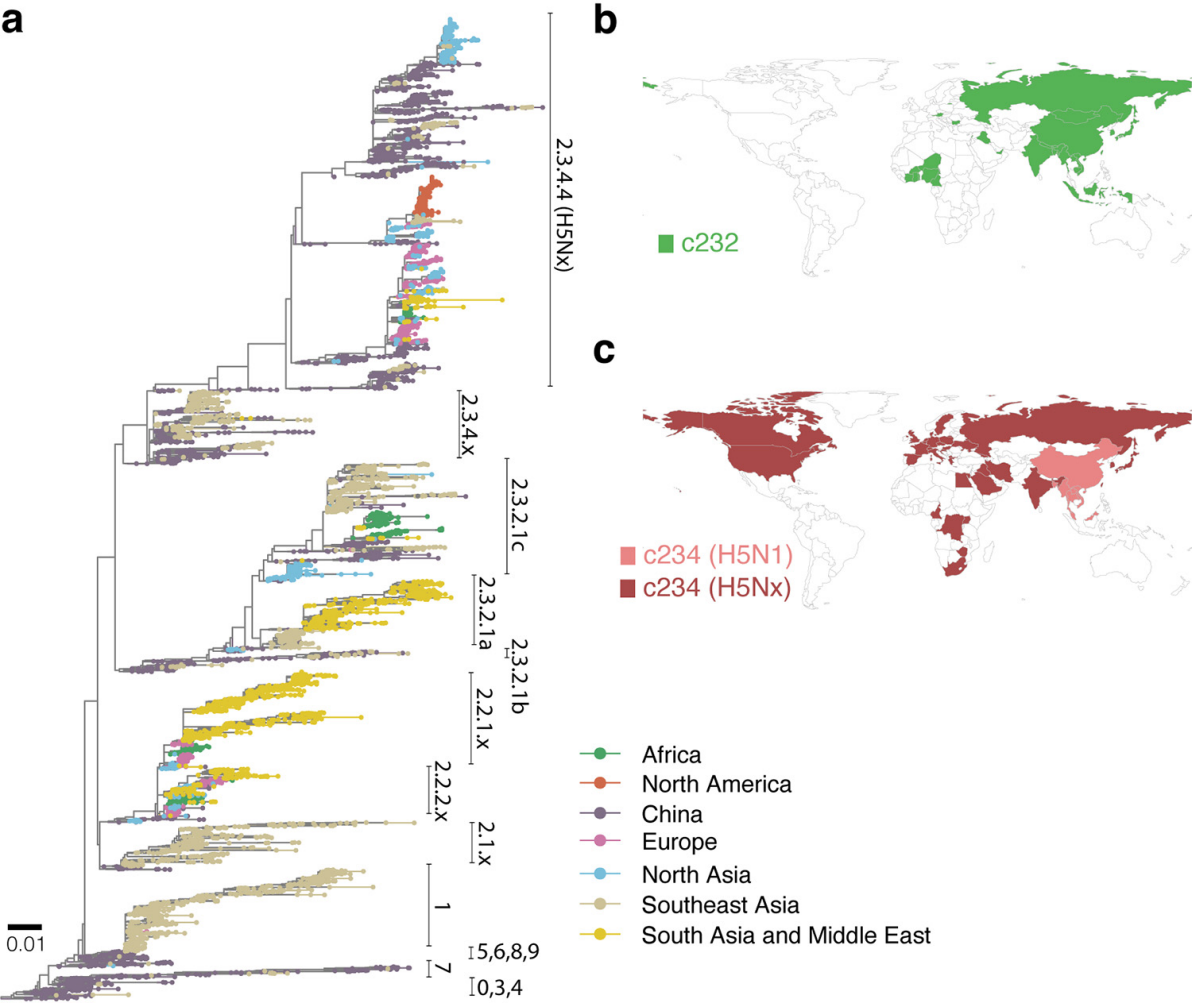
Geographical distribution of A/goose/Guangdong/1/96-like (Gs/GD) viruses based on available HA sequences. (a) Maximum likelihood tree was inferred with HA sequences of Gs/GD viruses with tips colored based on the location of isolation (*n* = 8,487). Different sublineages classified by the H5 nomenclature system are labeled next to the tips. Scale bar represents 0.01 substitutions per nucleotide site. Global distributions of clade 2.3.2 (b) and H5N1 and H5Nx clade 2.3.4 (c) viruses are shown in pink and dark red, respectively.

Influenza A virus has a wide host range and is able to infect and transmit between diverse animals, including both birds and mammals (1). Highly pathogenic avian influenza (HPAI) A/Goose/Guangdong/96 (Gs/GD) H5N1 viruses have caused recurrent outbreaks in poultry and wild birds since they were first recognized in southern China in 1996 (2). After the successful establishment of Gs/GD viruses in China’s enormous domestic poultry population, the virus spread throughout Eastern and Southeast Asia, resulting in devastating economic losses as a consequence of infection or culling of infected poultry (3). H5N1 viruses subsequently underwent diversification (Fig. 1a) and are classified into multiple clades that are often not antigenically cross-reactive, greatly complicating vaccination strategies (3, 4). Reverse spillover of H5N1 into migratory birds occurred at Qinghai Lake in 2005 (5), leading to intercontinental spread of the virus to the Middle East and Europe (6). Since then, many studies have focused on the distinct roles played by avian species in different ecological niches in virus dispersal (7–9). As of May 2020, 861 human H5N1 cases and 455 fatalities have been recorded (10).

Until 2014, outbreaks in poultry and human infections caused by Gs/GD viruses were exclusively H5N1 subtype (11, 12). H5N1 underwent frequent reassortments with low-pathogenic avian influenza (LPAI) viruses carrying different neuraminidase (NA) subtypes but only acquired internal genes to form distinct genotypes (13, 14). In contrast, Gs/GD isolates possessing an NA gene other than N1 were infrequently detected (11, 12), with H5 genes generally coevolving with N1 genes within the Gs/GD sublineages (13). However, since 2014, a significant change in the NA subtype occurred with the emergence of reassortant H5Nx virus outbreaks in poultry (15, 16). These H5Nx viruses include H5N2, H5N6, and H5N8 subtypes initially identified in China and Northern Asia that subsequently spread to different regions across Asia, Europe, and North America, far exceeding the distribution of H5N1 viruses (Fig. 1b and c). These H5Nx viruses demonstrated unprecedented compatibility with different NA subtypes, rapidly generating novel H5-NA combinations with endemic strains when introduced to other countries (15, 17). It is unclear what factors may have driven the transition from a single avian N1 subtype to multiple non-N1 NA subtypes.

## RESULTS

### Evolution and coevolution of diverse H5 clades in China.

**FIG 2:**
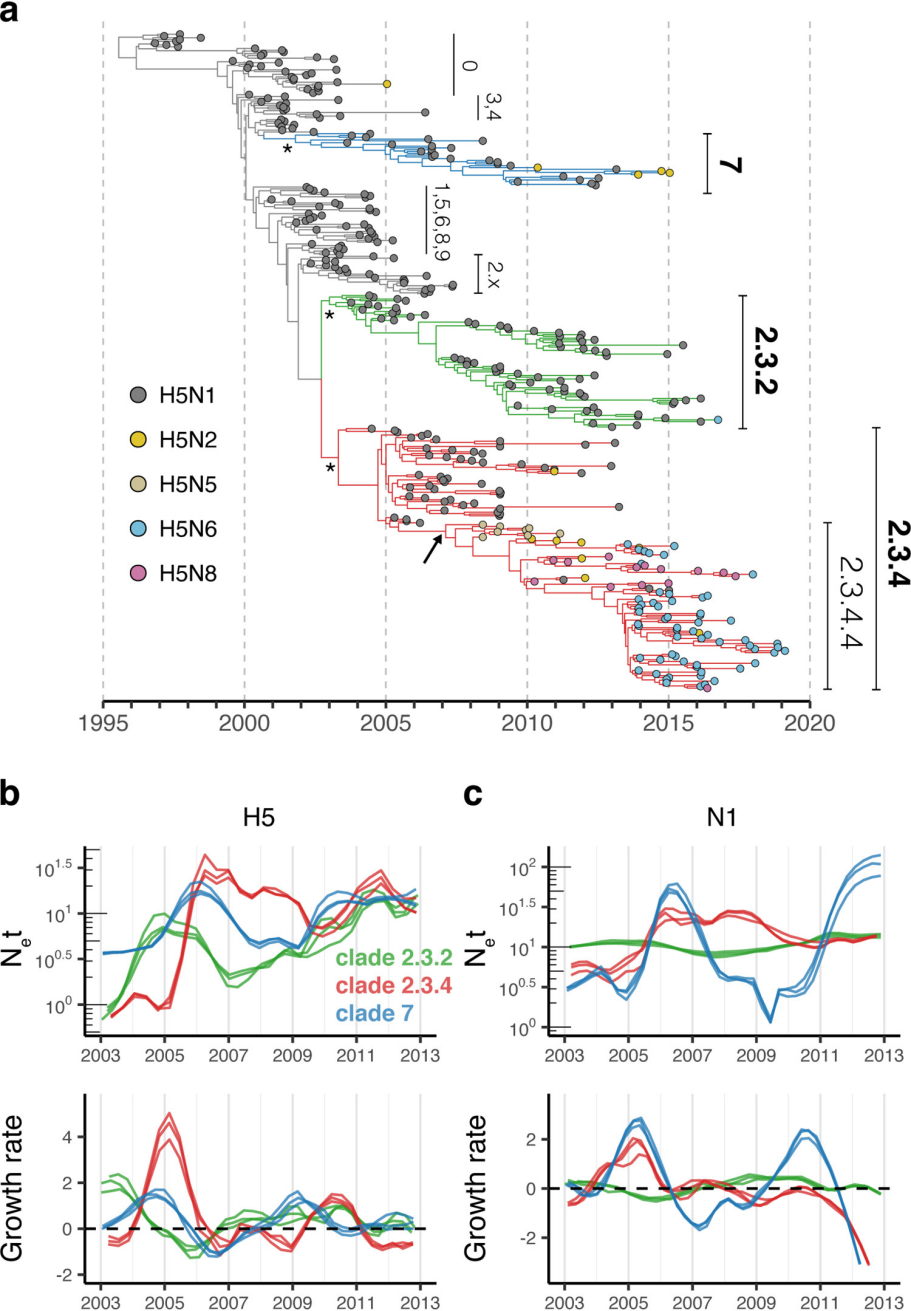
Phylogeny and population dynamics of Gs/GD viruses in China. (a) Maximum clade credibility (MCC) tree was reconstructed using HA genes from viruses isolated in China (*n* = 369). Tips are colored according to their subtypes. Different sublineages classified by the H5 nomenclature system are labeled next to the tips. Three major clades (2.3.2, 2.3.4, and 7) are labeled in bold, with their times of most recent common ancestors (tMRCAs) highlighted by asterisks. tMRCA of clade 2.3.4.4 (H5Nx) is indicated by an arrow. Bayesian Skygrid method was used to infer effective population sizes (N_e_t) using H5 (*n* = 122 to 123, 201, and 155 for clades 2.3.2, 2.3.4, and 7, respectively) (b) and N1 genes (*n* = 135, 88, and 35 for clades 2.3.2, 2.3.4, and 7) (c). Corresponding growth rates of population size were estimated by skygrowth method using annotated MCC trees generated in the same Bayesian run. Results of three randomly sampled data sets are shown. N1 sequences belonging to clade 2.3.4.4 viruses were not included in the analyses. Horizontal dashed line in the growth rate panels indicates zero.

To better understand the mechanisms underlying the emergence of H5Nx viruses, we first characterized overall H5 virus diversity in China. By the early 2000s, H5N1 viruses were highly prevalent in poultry in China with spillover to wild birds (18), and three major monophyletic clades (clades 2.3.2, 2.3.4, and 7) had become predominant (Fig. 2a), constituting >50% of all available H5-hemagglutinin (HA) sequences in GenBank and GISAID databases from 2005 to 2019 (see Fig. S1a in the supplemental material). Other H5N1 clades were undetected from 2008 onward. With temporal phylogenies of H5-HA genes, we inferred the time of the most recent common ancestors (tMRCAs) of clades 2.3.2, 2.3.4, and 7 as 2002, 2003, and 2001 (Fig. 2a), indicating that they cocirculated for more than 10 years. The first detected clade 2.3.4.4 H5Nx virus was H5N5 isolated from a domestic duck in China in 2008 (19), which was >1 year divergent from the most closely related clade 2.3.4 H5N1 virus (Fig. 2a), indicating H5Nx viruses circulated undetected in that period. H5Nx viruses subsequently became predominant in China, eventually replacing their ancestral H5N1 viruses (Fig. 2a; see also Fig. S1b). The tMRCAs of clades 2.3.4.4 and 7 H5Nx viruses are both estimated as early 2007 (March 2007) with an uncorrelated rate model (Table 1), suggesting that they emerged during the same period. The concordant emergence of H5Nx viruses in multiple clades represents a major change in the evolutionary landscape of the Gs/GD H5N1 lineage. H5Nx viruses subsequently spread from China to multiple countries, including the first incursion of Gs/GD viruses to North America (Fig. 1a and c). These results show the dynamic lineage replacement occurring in China that corresponded with H5Nx emergence.

**TABLE 1:** Estimated dates of nodes within the H5-HA phylogeny as shown in Fig. 2a

Model combinations	tMRCA (95% HPD^*c*^)		
	Clade 234 H5Nx (internal node^*a*^)	Clade 234 H5Nx (root^*b*^)	Clade 7 H5N2
Strict/Skygrid	2007.28 (2006.91–2007.61)	2006.91 (2006.33–2007.41)	2006.17 (2005.50–2006.85)
UCLN/Skygrid	2007.18 (2006.66–2007.63)	2006.91 (2005.89–2007.66)	2007.18 (2006.42–2007.93)

a tMRCAs were identified from MCC trees built by clade 2.3.4-N1 (i.e., clade 2.3.4.1 to 2.3.4.3) and clade 2.3.4.4.

b tMRCA were identified from MCC trees built using only clade 2.3.4.4 virus sequences.

c HPD, highest posterior density.

### Comparative population dynamics of H5 viruses.

To understand virus population behavior prior to the emergence of H5Nx viruses, we compared temporal changes in population sizes and growth rates of clades 2.3.2, 2.3.4, and 7. We first used the HA genes to infer effective population size using the Skygrid model implemented in a Bayesian phylogenetic framework (20, 21). The population size of all three clades (2.3.2, 2.3.4, and 7) increased exponentially to peak during 2005 to 2006, followed by an approximate 0.5 times contraction in population size, which recovered to their original levels after 4 to 5 years (Fig. 2b; see also Fig. S2a and S3 in the supplemental material). The growth rate estimates reflect the same population dynamics, with positive rates occurring until 2005 when clade 2.3.2 rates dropped below zero, followed by negative growth rates for clades 2.3.4 and 7 in 2006, indicating a contraction in population size. These negative growth rates matched the reduction in population size and remained low for subsequent years until 2010 (Fig. 2b). The patterns of population dynamics by the NA-N1 gene (Fig. 2c; Fig. S2b) demonstrated some distinguishing characteristics. For instance, the NA-N1 of clade 2.3.2 viruses had a relatively constant population size, but fluctuations were observed for clades 2.3.4 and 7. In clade 7, the HA and NA both showed a population decline following a peak in early 2006. Although the population contraction observed in the clade 2.3.4 HA gene was less obvious for the NA gene, growth rates of clades 2.3.4 and 7 both dropped from mid-2005 to 2007, the same pattern shared by HA estimates for all three clades (Fig. 2b). The decline of clade 2.3.4 after 2009 and the dramatic reduction in clade 7 inferred by NA-N1 are associated with the emergence of non-N1 reassortant viruses (Fig. 2c; Fig. S1b). Consistent patterns in population dynamics were present across the three replicates of these analyses (Fig. 2b and c; Fig. S2) regardless of subsampling (Fig. S3). We observed discordant patterns of population size and growth rates between HA and NA genes, which are likely due to the effect of interclade reassortment, as frequent reassortment events are common among clades, especially clade 2.3.2 (see Fig. S4 in the supplemental material). Notably, the emergence of clade 2.3.4.4 and 7 H5Nx viruses in early 2007 (Table 1) occurred after the observed population contraction and shows that they survived the bottleneck.

Comparative analysis of a contemporaneous H6 virus lineage (A/wild duck/Shantou/2853/2003-like) (22, 23), which is highly prevalent in poultry in China, showed no comparable bottleneck during 2005 to 2011 (see Fig. S3 and S5 in the supplemental material), suggesting that these population declines were not uniform across all avian influenza subtypes in China. To further test the effect of sampling, we arbitrarily created a sampling scheme in which no sequences were isolated in 2007 plus only half of 2008 sequences were selected compared to that in other sampling years. Results from this artificial scheme show a drop in population size lasting for less than 2 years with a magnitude smaller than 0.2 times (Fig. S3 and S5), suggesting the observed H5 clade population dynamics in China were not easily shaped by temporal absence of sampling effort or sequence availability.

We then performed neutrality tests with the HA nucleotide sequences to investigate population dynamics independent of Bayesian coalescent-based methods. Tajima’s D is a scaled comparison of the genetic diversity and the number of segregation sites in the sequences (24). When more rare alleles are found in the samples, which may result from population expansion or selection sweep, Tajima’s D would be negative. Conversely, when fewer low-frequency alleles are found, which may result from ongoing population bottleneck or balancing selection, Tajima’s D would be positive (24, 25). Our results indicate that Tajima’s D values of clades 2.3.2, 2.3.4, and 7 viruses all showed an upward trajectory from 2008 to 2010 and were generally negative outside of that window (see Fig. S6 in the supplemental material). These results suggest a dramatic reduction in excess low-frequency alleles occurred in H5N1 during 2008 to 2010, supporting the bottleneck inferred by the Bayesian coalescent-based methods (Fig. 2b). Furthermore, the Tajima’s D values of all three clades were generally consistent with Fu and Li’s D (Fig. S6b) and Fu and Li’s F (Fig. S6c), two derivatives of Tajima’s D following similar principles but taking into account singleton sites instead of segregation sites (26). These results suggest a demographic change in H5N1 viruses in China prior to the emergence of H5Nx viruses.

### Ecosystem interactions and transmission dynamics of H5 virus.

**FIG 3:**
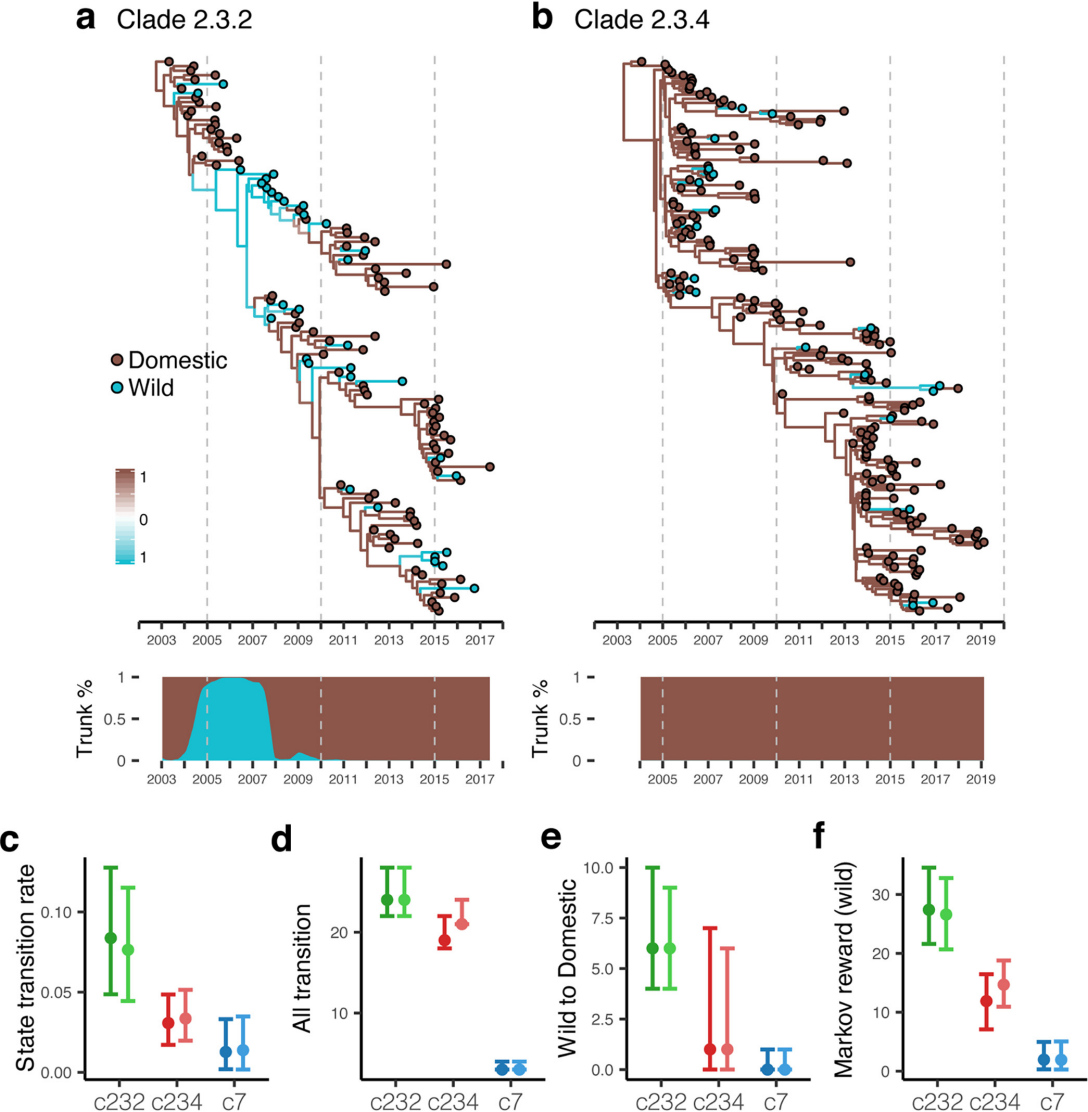
Gene flow of Gs/GD clades between ecological systems in China. MCC trees were reconstructed using HA genes of clade 2.3.2 (*n* = 123) (a) and 2.3.4 (*n* = 201) (b) viruses. Tips are colored according to their assigned ecological states, while internal branches are colored according to ancestral states inferred using a Bayesian phylogenetic framework. The shaded boxes indicate posterior probability values for both ecological states on the phylogenetic trees. Trunk proportions of phylogenetic trees occupied by the two ecological states summarized by PACT are shown below the trees. Overall transition rates (c), total counts of Markov jump (d), Markov jump from wild to domestic states (e), and Markov reward in the wild states (f) were estimated from the same Bayesian analyses conducted in panels a and b and also for clade 7 (see Fig. S7 in the supplemental material). Results using another random sampled data set allowing for greater sample sizes for each clade are shown in lighter shades. Error bars represent 95% highest posterior density (HPD) intervals.

To understand whether agricultural activities influenced virus population size, we investigated H5N1 virus transmission between domestic and wild ecosystems in China. The rationale here is that, for example, increasing poultry production would boost the virus population, while control measures such as culling or vaccination would have the opposite effect, and virus populations in wild birds would be largely unaffected by human activities. To achieve this, we integrated the ecosystem type (i.e., domestic versus wild host populations) into an ancestral state reconstruction analysis and estimated the rate of viral transmission between domestic and wild ecosystems using a Bayesian phylogenetic diffusion model (27). Our results indicate that domestic-to-domestic transmissions dominated during the early diversification of clade 2.3.2, 2.3.4, and 7 viruses in China (Fig. 3a and b; see also Fig. S7 in the supplemental material). However, some marked differences between these virus clades were observed. From 2005 to 2007, clade 2.3.2 viruses showed noticeable transition from circulation in domestic to wild hosts, with the wild ecosystem reaching 100% of the trunk proportion in 2006, although subsequent transmissions were predominantly mediated by domestic-domestic spread (Fig. 3a). In contrast, clade 2.3.4 and 7 viruses showed limited cross-ecosystem transmission, and the domestic ecosystem occupied the trunk across their phylogenies (Fig. 3b; Fig. S7). Distribution of the ecological source of virus sequences in public databases also reflected the inferred trunk proportions, notably with over half of clade 2.3.2 viruses isolated from wild animals during 2007 to 2009 (Fig. S1c). Interestingly, we found that the transition rates (Fig. 3c) of clade 2.3.2 viruses were significantly greater than those of clade 2.3.4 and clade 7 viruses, reflecting more frequent interecosystem virus flow within clade 2.3.2. The total number of state transitions were higher in clade 2.3.2 and clade 2.3.4 but lower in clade 7 virus (Fig. 3d); however, only clade 2.3.2 had a greater number of state transitions from wild to domestic ecosystems (Fig. 3e). This is also reflected by the Markov rewards (Fig. 3f), which indicate that clade 2.3.2 viruses have circulated in wild birds significantly longer (∼27 years) than clades 2.3.4 and 7 viruses (∼12 and ∼2 years, respectively). Analysis of a larger H5-HA data set indicated that these results were not significantly affected by sample size. As such, clade 2.3.2 viruses demonstrated more extensive circulation in wild birds and more readily transmitted from wild birds to poultry. Overall, our analyses show that the wider ecological distribution of clade 2.3.2 viruses shielded them from the H5N1 virus population declines in poultry that led to the replacement of N1 viruses in clade 2.3.4 and 7 with non-N1 viruses (Fig. 2a; Fig. S1b).

## DISCUSSION

In this study, we analyzed the population dynamics of the three major HPAI H5N1 virus sublineages in China. We show that the first non-N1 Gs/GD H5 viruses, i.e., H5Nx viruses, emerged after population bottlenecks were observed in H5N1 viruses, indicating that H5N1 population declines did not result from competition with H5Nx viruses. A scenario involving competitive exclusion entails limited host population and cocirculation for an extended duration (28), which is not the case considering the abrupt change in population size that we observed (Fig. 2) and the tremendous poultry population in China (see Fig. S8b in the supplemental material). We further show that clade 2.3.2 viruses were subject to a less severe population decline that is attributable to their circulation in wild bird populations during the bottleneck period, and that H6 viruses in poultry were not subject to any population bottleneck. Together, this data suggests that the implementation of comprehensive H5N1 control strategies in China in 2005 (11, 29) successfully suppressed H5N1 viruses in poultry directly before the emergence of H5Nx viruses.

Vaccination and aggressive culling (11, 29, 30) successfully reduced the number of H5 outbreaks in China until the emergence of H5Nx viruses (Fig. S8a) despite stable poultry production numbers (Fig. S8b) (31). Chinese national surveillance data (11) subsequently showed dramatic changes in the proportion of H5N1 virus clades detected in poultry that matches the results of our analysis. Specifically, in 2007, clade 2.3.4 viruses accounted for 100% of H5N1 viruses but fell to <35% from 2008 to 2013 before rebounding to over 92% from 2014 to 2018 (11). In contrast, the proportion of clade 2.3.2 viruses went from zero in 2007 to >51% from 2008 to 2013 before falling to <7% from 2014 to 2018. These dynamics are further supported by the absence of H5N1 in poultry markets in Hong Kong from 2004 to 2008, although viruses were isolated in wild birds (32). We therefore hypothesize that the widespread suppression of the H5N1 virus population in poultry provided susceptible hosts in which antigenically distinct H5Nx viruses (33, 34) could propagate and emerge. Recombinant H5N1 vaccines produce potent anti-HA and anti-NA antibody responses in chicken and other poultry (35, 36) but do not provide sterilizing immunity (37–39). The widespread use of H5N1 vaccines would therefore confer a fitness advantage to H5Nx viruses due to the antigenic mismatch of the NA. However, we cannot discount the possibility that H5Nx viruses were fixed stochastically in the H5 virus population due to the strong genetic drift caused by the observed bottlenecks (40).

Our study stresses the importance of intensified monitoring of pathogen populations during the application of control measures, particularly widespread vaccination programs that have the potential to lead to the emergence of antigenic escape (41, 42). This includes continued surveillance of related viruses in wildlife that may act as a reservoir for the reemergence of those pathogens (5, 43, 44). Vaccination programs against avian influenza viruses would benefit from universal vaccines that target a wider diversity of viruses, preventing the potential emergence of novel subtypes residing in poultry or wild bird populations.

## MATERIALS AND METHODS

### Data source and preparation.

H5 and H6 hemagglutinin (HA) and associated neuraminidase (NA) sequences of avian influenza A viruses were downloaded from NCBI Influenza Virus Resource (https://www.ncbi.nlm.nih.gov/genomes/FLU/) and GISAID (https://www.gisaid.org) on 15 January 2020. Sequences downloaded were subjected to the following search criteria: (i) each viral strain required both HA and NA sequences, (ii) sequences had known host information, and (iii) lab-derived strains and mixed subtypes were excluded. Short sequences (0.95× the lengths of coding region) or sequences containing more than 5% ambiguous nucleotides were then removed. Curated data sets were aligned using MAFFT v7.407 (45) and trimmed to coding regions and additional multiple basic amino acids at the H5 cleavage site also removed.

### Preliminary phylogenetic analyses and data set design.

A maximum likelihood (ML) tree was inferred for the HA and NA alignments using IQ-TREE v1.6.12 (46) with a general time reversible (GTR) substitution model plus a gamma-distributed rate. Outliers generated by mislabeling or sequencing errors were detected using TempEst v1.5.3 (47) and discarded. Viral sublineages defined by HA genes, or clades, were classified according to World Health Organization Gs/GD H5N1 nomenclature (12) or previous studies of H6 viruses (22, 23). We define clades to include viruses in all descendant subclades, e.g., clade 7 includes viruses in clades 7.1 and 7.2. Due to their monophyletic relationship and small sample sizes, clade 2.3.1 and clade 2.3.3 viruses were combined with clade 2.3.2 and clade 2.3.4, respectively (12).

Identical sequences were removed from the analyses prior to any subsampling. To obtain an amenable number of sequences for Bayesian analyses and to avoid biases made by oversampled sequences presented by distinct outbreaks (48), we subsampled both the whole Gs/GD lineage and each clade uniformly with at most 25 sequences per year. Sequences isolated in the same animal or that clustered spatially were grouped, and a single representative sequence was randomly chosen in each year. HA sequences of H5 reference strains were included to maintain the topology of phylogenetic trees (12). From the total available clade 2.3.2 (*n* = 276), clade 2.3.4 (*n* = 1,085), and clade 7 (*n* = 74) nonredundant HA sequences in China, we produced subsampled data sets that contained 122 to 123, 201, and 55 sequences for the three clades, respectively. Likewise, a total of 250, 174, and 51 nonredundant N1 sequences in China for clades 2.3.2, 2.3.4, and 7, respectively, were subsampled to data sets of 135, 88, and 35 sequences. Larger data sets, containing up to 30 HA sequences per year, were used for the cross-ecosystem diffusion analyses; this sampling scheme increased the size of the clade 2.3.4 data set (*n* = 223) but not the sizes of data sets for clades 2.3.2 and 7. To examine the possible effect of uneven sampling efforts that may be present in surveillance data, we generated an H6 data set using an artificial sampling scheme in which no sequences were sampled in 2007 and only 15 sequences were selected in 2008 compared to up to 30 sequences being sampled in other years. The discontinuously sampled data sets contain 184 H6 sequences sampled from 535 nonredundant A/wild duck/Shantou/2853/2003-like sequences in China; the size is comparable to the typical data sets of H6 sequence generated by the methods applied to H5/N1 genes (*n* = 182).

### Population dynamics.

Changes in viral population size over time were estimated using the Skygrid demographic model (21) with an SRD06 nucleotide substitution model (49) and uncorrelated lognormal relaxed molecular clock (50) as implemented in BEAST v1.10.4 (20). For taxa without an associated day or month, date samplings were applied from uniform distributions within the known temporal bounds. Markov chain Monte Carlo (MCMC) analysis was run for 120 million steps and sampled every 10,000 steps. Tracer v1.7.1 (51) was used to inspect convergence of parameters (ESS values of >200) after discarding the first 20 million steps per MCMC chain. We tested the fit of the Skygrid model against a constant coalescent prior to our data sets by comparing the marginal likelihood using both path-sampling and stepping-stone approaches (52, 53) and found the Skygrid prior provided a better fit for all data sets (see Table S1 in the supplemental material). Maximum clade credibility (MCC) trees were summarized using TreeAnnotator (20). The annotated MCC trees were also used with the skygrowth model to calculate growth rates (54). To test the robustness of the estimates, time of the most recent common ancestor (tMRCA) of H5Nx was acquired in the internal node of the MCC tree containing all HA genes of clade 2.3.4 and from the root height of the MCC tree containing only HA genes of clade 2.3.4.4 (H5Nx). To obtain the tMRCA of clade 7 viruses, an arbitrary taxa group was set to include all H5N2 viruses in each data set. Combinations of either a strict or a relaxed molecular clock model were employed to further test the robustness of tMRCA estimates.

### Discrete trait transition analyses.

For analyses on the distribution of virus ecology, we classified each sequence as either wild or domestic according to strain name and information provided in the original database entry or publication. The sequences isolated from poultry (e.g., duck, chicken, goose) and human infections were classified as domestic state. For other animal species and environmental samples, their ecological states were designated based on the location of sample collection (see Fig. S1 in the supplemental material or source data at https://github.com/yaotli/n1tonx for these ecological states). Diffusion patterns between the two ecosystems were inferred using a discrete-state continuous time Markov chain (CTMC) model implemented in BEAST (27). We employed an asymmetric substitution model with Bayesian stochastic search variable selection (BSSVS) for transition parameters (27). Equal probability of the two states were assigned to sequences that were unable to be classified based on available information as described in a previous study (55). Number of transitions (Markov jumps) between the two states and the time spent in each state (Markov rewards) along the branches were recorded during MCMC processes (56, 57). Priors used in the diffusion analyses were identical to those in the Skygrid analyses. A subset of 1,000 posterior trees from each MCMC process was resampled by LogCombiner (20), and PACT v0.9.4 (https://github.com/trvrb/PACT) was utilized to summarize trunk proportion occupied by the two states. Trunks were defined as ancestral branches shared by sequences isolated within 0.1 year of the most recent samples. All trees were visualized with ggtree (58).

### Population genetics statics.

Tajima’s D (24) and the derivative Fu and Li's D and F (26) were calculated using HA sequences isolated in every two consecutive years for each clade. We applied Fu and Li’s D and F methods that are estimated without an outgroup in this study (i.e., D*, F*) (26). The 2.5% and 97.5% percentiles were calculated from 500 bootstrapped samples.

### Data and code availability.

Custom codes used in the data set preparation, details of state designation, and XML files required for BEAST are available at https://github.com/yaotli/n1tonx. Accession numbers of sequences analyzed in our study are listed in Table S2 in the supplemental material.
