# Supplementary material for: H5Nx Viruses Emerged during the Suppression of H5N1 Virus Populations in Poultry
Source: Microbiol Spectr. 2021 Sep 29;9(2):e01309-21. doi: 10.1128/Spectrum.01309-21 (PMC8557938; doi:10.1128/Spectrum.01309-21)
Supplement: SUPPLEMENTAL FILE 1 — Supplemental material. Download SPECTRUM01309-21_Supp_1_seq2.pdf, PDF file, 3.1 MB [file spectrum01309-21_supp_1_seq2.pdf]

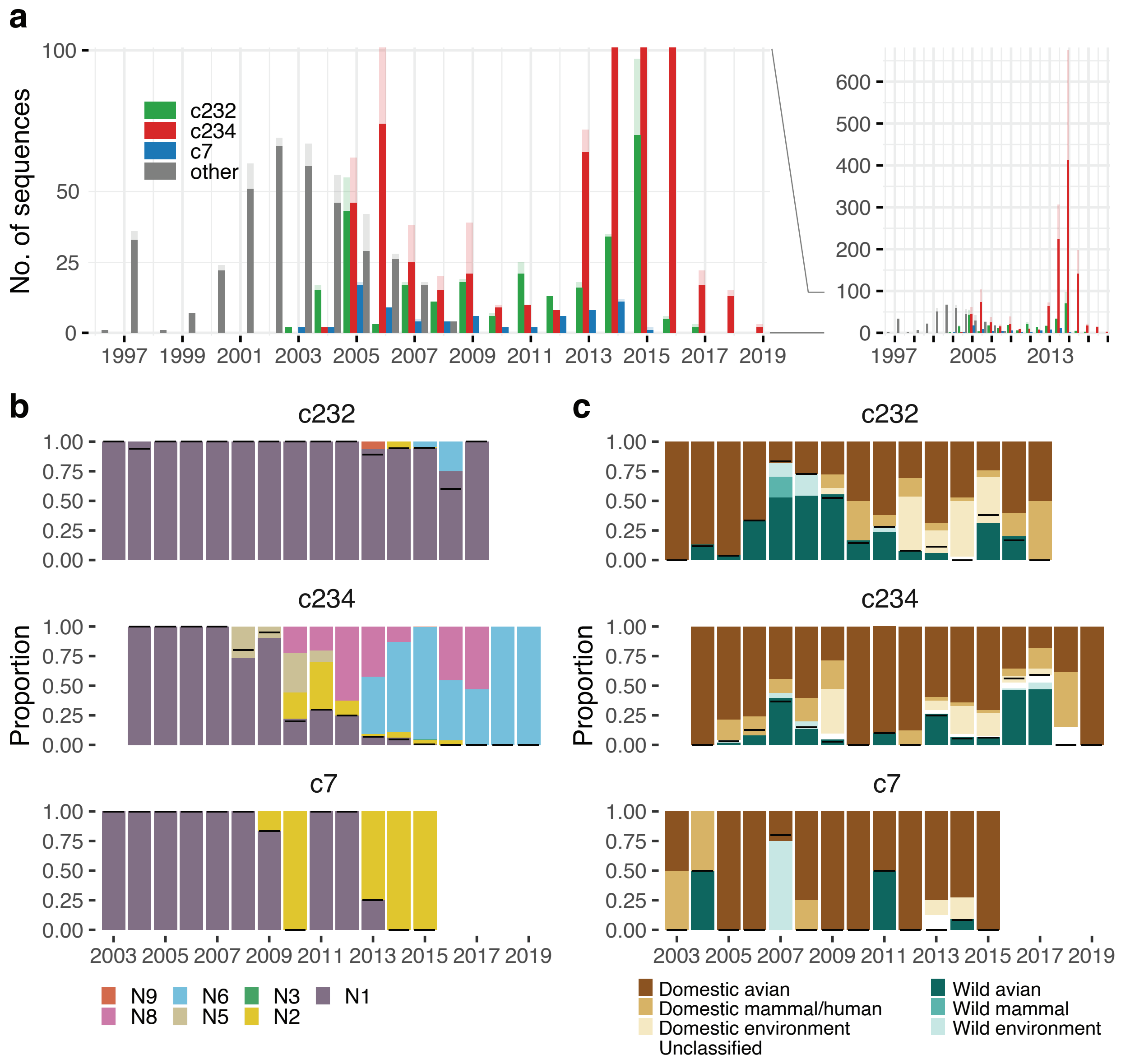

Supplementary figure 1. Characterization of GsGD viruses in China based on available H5-HA sequences. (a) Distribution of sequences amongst different H5 clades in China. Bars colored with light shade indicate numbers from all available sequences (n=2408), whereas colors with normal shade indicate numbers from a non-redundant dataset with identical sequences eliminated (n=1800). (b) Distribution of subtype of viruses in the non-redundant dataset (n=276, 1085 and 74 for clade 2.3.2, 2.3.4 and 7, respectively). Proportions of N1 subtype calculated using the redundant dataset are indicated by horizontal black bars in each column. (c) Distribution of virus sequences by ecosystem type. Horizontal bars indicate the sum of the three categories of wild ecological using redundant dataset (n=329, 1585 and 78 for clade 2.3.2, 2.3.4 and 7).

Supplementary figure 2

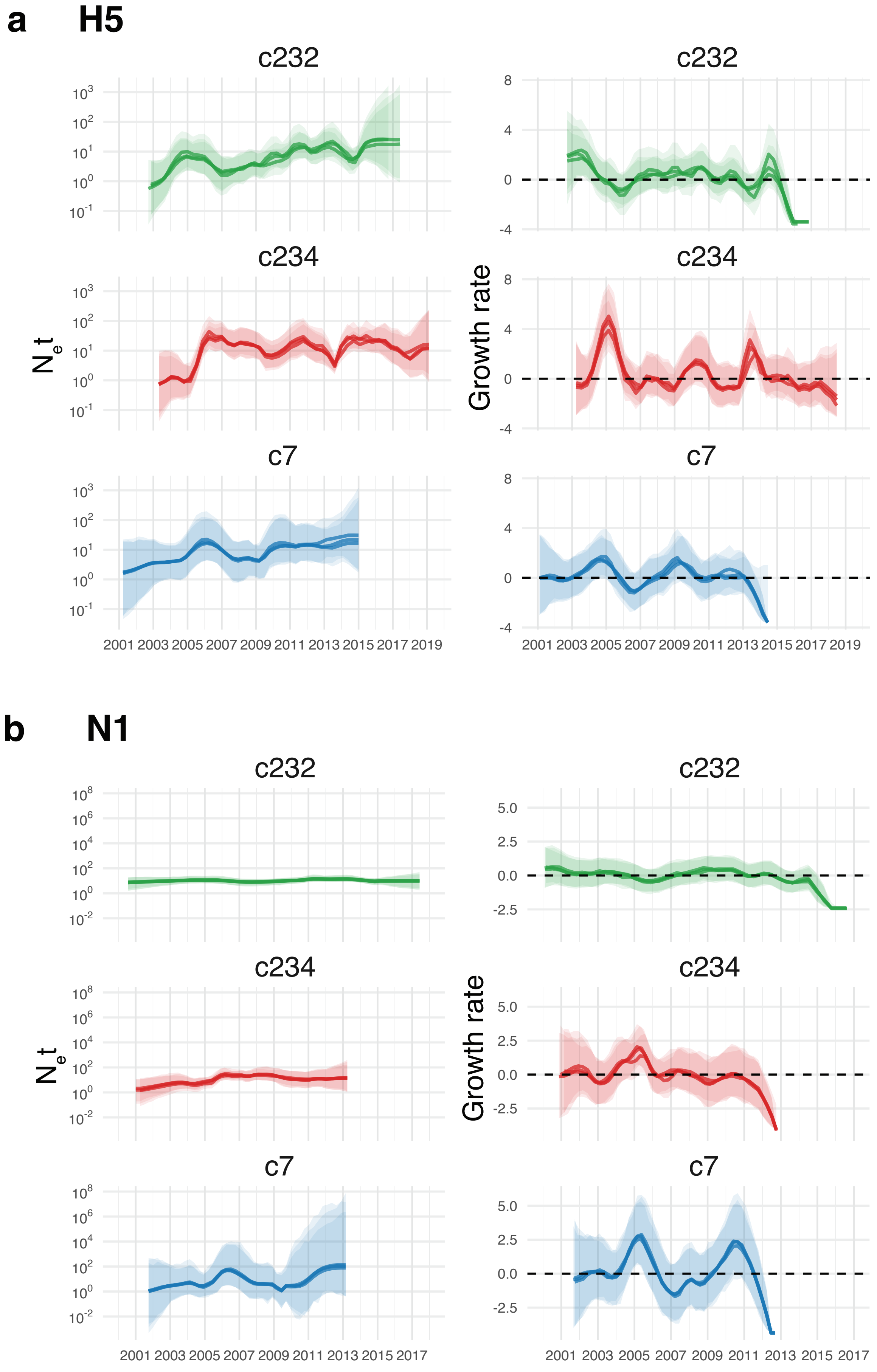

Supplementary figure 2. Population dynamics of the three GsGD clades in China. Effective population sizes ($N_e t$) and growth rates were calculated for the (a) H5 and (b) N1 genes using Bayesian Skygrid and Skygrowth methods, respectively. The sequence datasets were identical to those used in Figure 2. Shaded intervals indicate 95% highest posterior density (HPD) intervals in both analyses. Results of three randomly sampled datasets are shown for each clade. N1 sequences belonging to clade 2.3.4.4 viruses were not included in the analyses. Horizontal dashed line in the growth rate panels indicates zero.

Supplementary figure 3

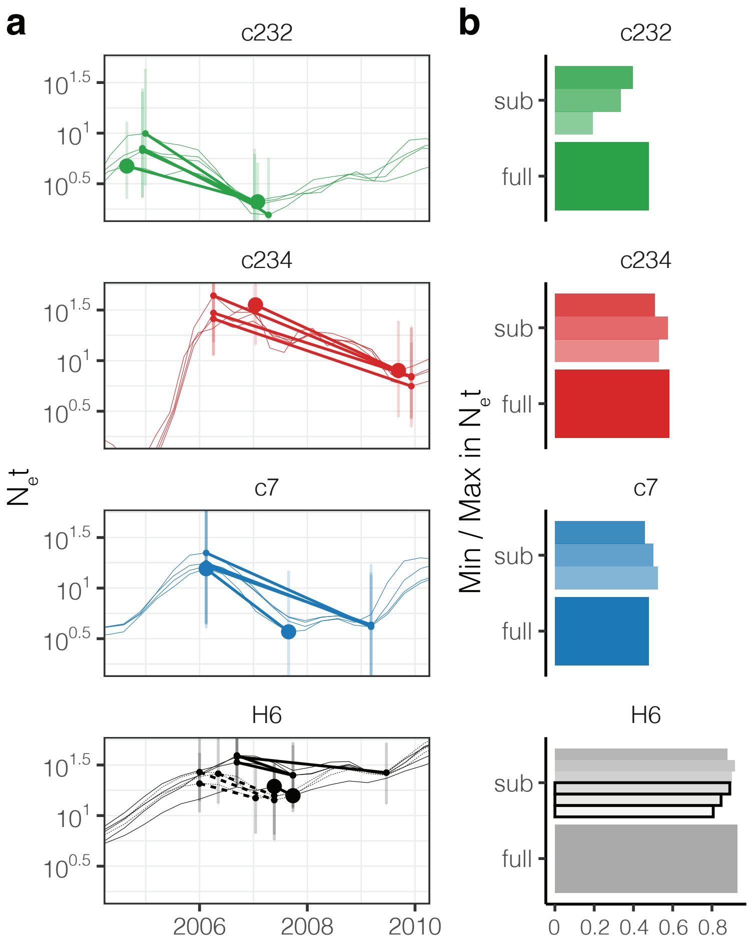

Supplementary figure 3. Sensitivity of subsampling to Skygrid analyses. (a) Population dynamics calculated with the full datasets were overlaid with results calculated by subsampling datasets. Differences between the peak before 2008 and the lowest point afterwards are highlighted, with larger points indicating full datasets. Outcomes of the discontinuous sampling scheme implemented in the H6 viruses are indicated by dashed lines. The vertical lines represent 95% HPD. (b) Ratio of minimum over maximum $N_e t$ value highlighted in panel (A) for each dataset. Bars with solid edges for H6 viruses indicate the discontinuous sampling scheme. Number of sequences in full datasets: 276, 500, 74 and 535 for clade 2.3.2, 2.3.4, 7, and ST/2853/03-like H6 lineages, respectively. The full dataset of clade 2.3.4 here does not include viruses isolated post 2014.

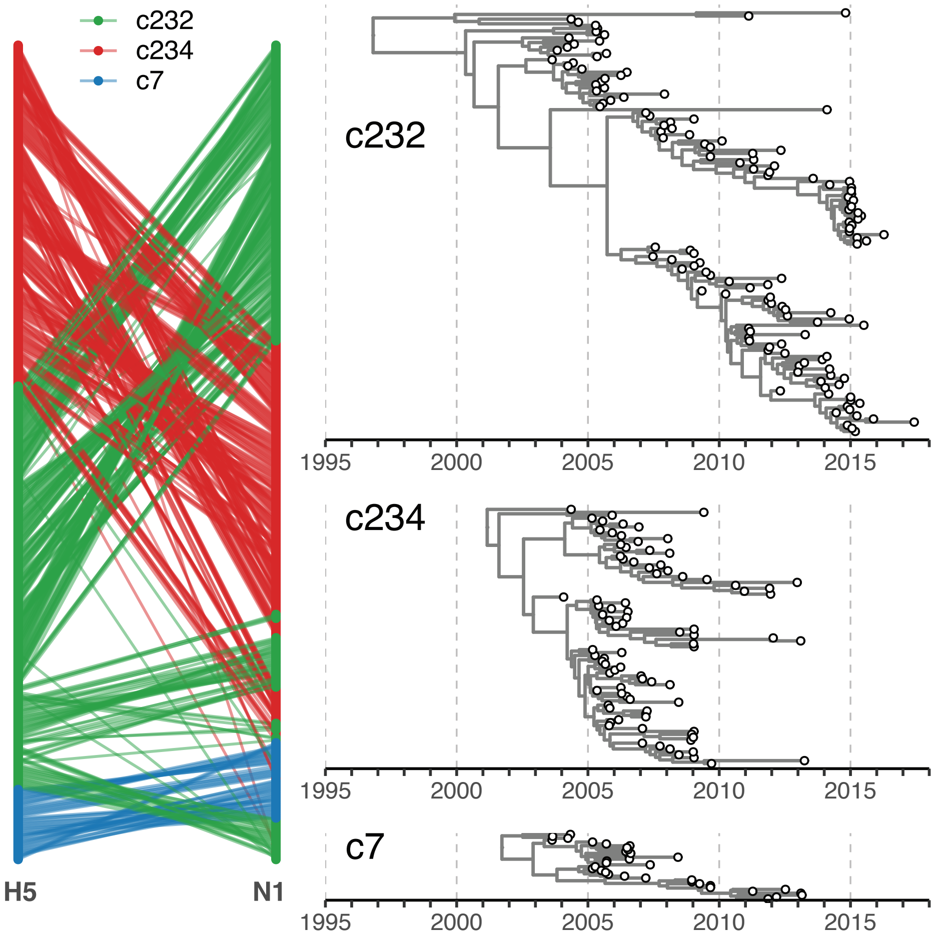

Supplementary figure 4. Coevolution of H5 and N1 genes of GsGD viruses in China. Comparison of phylogenetic relationships of the HA and NA genes by linking their location on the maximum likelihood (ML) trees generated using all available H5 and N1 avian influenza sequences (H5, n=8487; N1, n=5232) (left panel), where each line links the phylogenetic position of the HA and NA genes of the same virus. MCC trees (right panel) were reconstructed from one of the subsampled N1 datasets for each clade (n=135, 88, 35 for clade 2.3.2, 2.3.4 and 7, respectively). N1 sequences belonging to clade 2.3.4.4 viruses were not included in the analyses.

Supplementary figure 5

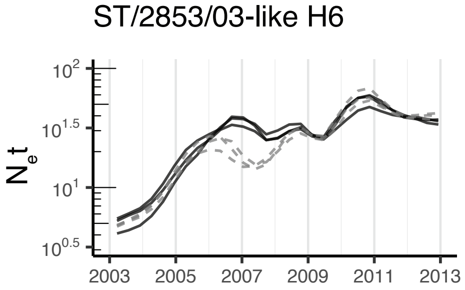

Supplementary figure 5. Population dynamics of A/wild duck/Shantou/2853/2003-like H6 viruses in China. Bayesian Skygrid method was used to infer effective population size ($N_e t$) using H6 genes (n=182). Gray dashed lines show the results using an artificial sampling scheme in which no sequences were sampled in 2007 and only 15 sequences were selected in 2008 compared to up to 30 sequences being sampled in other years (n=184). Results of three randomly sampled datasets are shown for each sampling scheme.

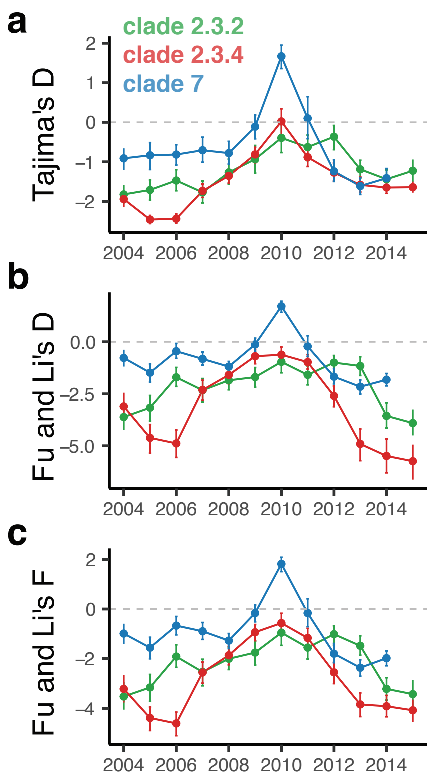

Supplementary figure 6. Tests of constant population size based on nucleotide polymorphism. (a) Tajima's D, (b) Fu and Li's D, and (c) Fu and Li's F were calculated using HA sequences of the three clades (n=276, 1085 and 74 for clade 2.3.2, 2.3.4 and 7, respectively). Each time point was calculated with sequences isolated in that year in addition to sequences from the following one year. Error bars represent 95% bootstrap percentiles.

Supplementary figure 7

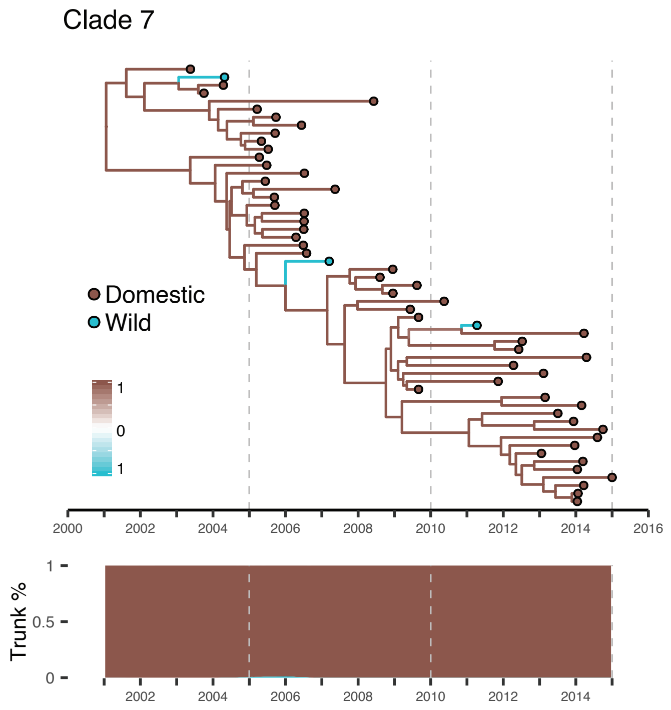

Supplementary figure 7. Gene flow between ecological systems for clade 7 HA genes in China (n=55). Tips are colored according to their assigned ecological states, while internal branches are colored according to ancestral states inferred using a Bayesian phylogenetic framework. The shaded box indicates posterior probability values for both ecological states on the phylogenetic trees. Trunk proportions of phylogenetic trees occupied by the two ecological states summarized by PACT are shown below the tree.

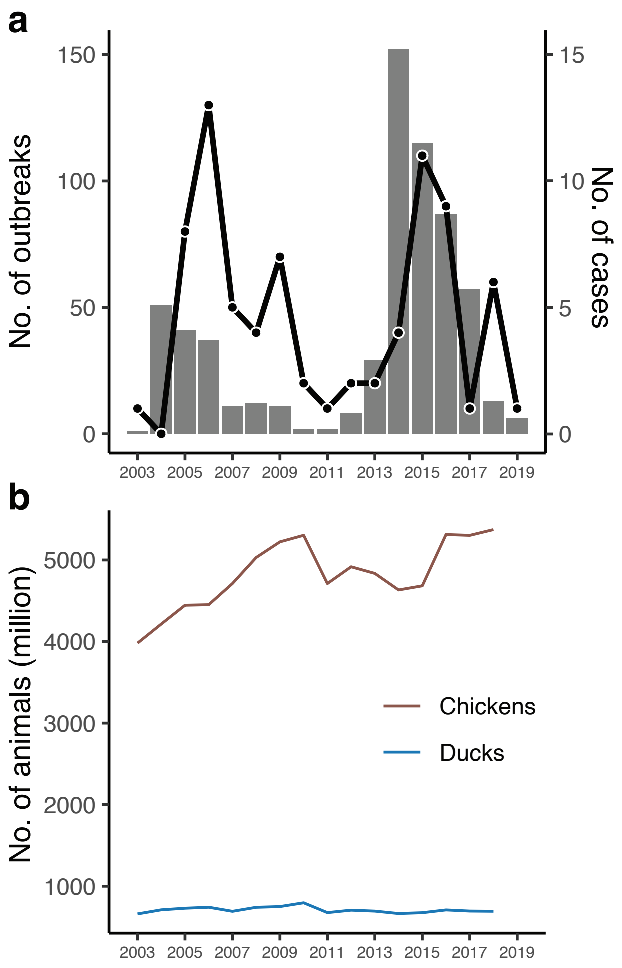

Supplementary figure 8. Epidemiology of Gs/GD viruses and poultry production in China. (a) Numbers of HPAI H5 outbreaks (bar chart) and human infections with GsGD viruses (line) in China based on FAO and WHO data. (b) Poultry production numbers in China based on statistics from FAO.

Supplementary table 1. Model selection of coalescent priors

Dataset	Path Sampling		Stepping Stone	
	Constant	Skygrid	Constant	Skygrid
2.3.2	-10528	**-10515**	-10529	**-10517**
2.3.4	-17235	**-17181**	-17239	**-17188**
7	-8659	**-8653**	-8659	**-8652**

Log marginal likelihoods were calculated by path-sampling and stepping-stone methods for the two coalescent tree priors (constant vs Skygrid). The sampling was run for 50 path steps with a chain length of one million. The better fit model of each dataset was in bold.

Supplementary table 2. List of database accession numbers from GenBank and GISAID used in the genetic analyses for each gene phylogeny.

	H5 GsGD	H5 c232	H5 c234	H5 c7	N1 c232	N1 c234	N1 c7	H6
1	AB521159	AY518362	CY030905	DQ914814	AY518363	DQ835315	DQ914816	GU324774
2	AB557629	AY737289	EU329186	EF587277	AY737291	CY030891	EF587279	HM144506
3	AF036356	CY036245	FJ784851	EU430496	AY737299	EU329184	EU430497	HM144485
4	AF084279	CY036213	FJ784852	DQ343150	CY036239	EU434688	DQ349118	HM144473
5	AF084280	CY036229	CY036269	EU195408	CY036215	FJ784771	EU195410	HM144519
6	AF084532	CY036253	EU195400	GU182230	CY036231	CY036271	GU182248	HM144528
7	AF102676	GU182166	HM172086	HM172092	EU874900	FJ784775	HM172172	HM144509
8	AF144305	GU182206	HM172088	HM172113	GU182168	EU195402	HM172199	HM144496
9	AF364334	CY063318	HM172096	HM172078	GU182144	EU195394	HM172206	HM144492
10	AF468837	HM172116	HM172069	HM172077	GU182176	GU182184	HM172196	HM144472
11	AF509016	HM172105	HM172097	HM172076	CY098760	GU182216	HM172205	HM144488
12	AF509017	CY098758	HM172101	HM172073	JN646727	HM172170	HM172212	HM144515
13	AF509020	JN646713	HM172079	HM172082	JN646732	HM172202	HM172194	HM144529
14	AY059477	JN646714	HM172100	HM172106	JN646730	HM172203	HM172192	HM144505
15	AY075027	JF975561	HM172070	HM172081	JF975563	HM172209	HM172197	HM144490
16	AY221522	JQ638673	GU727653	HM172080	JQ638678	HM172191	HM172201	HM144522
17	AY518362	JX576788	GU727661	HM172091	JX576793	HM172177	HM172193	HM144493
18	AY575871	CY146644	GU727677	HM172090	CY146646	HM172174	JQ277227	HM144497
19	AY575876	CY146692	GU596984	JQ277225	CY146694	HM172188	EU243145	HM144498
20	AY575877	CY146708	CY098790	EU243133	CY146702	HM172200	KC683526	GU220599
21	AY585357	JX534589	CY098798	EU243146	CY146710	HM172168	JX534551	JF965139
22	AY585358	JX534581	CY098822	JQ041399	JX534591	GU727679	KX160195	JF965163
23	AY585359	JX534573	JX565019	KF150634	JX534583	GU596986	KX160180	JF965165
24	AY585360	KC357320	JQ041401	KC683522	JX534575	CY098808	MF116322	JF965147
25	AY585361	GU477547	JX507355	JX534549	JX534559	KC690158	HQ677024	JF965156
26	AY585362	KJ003987	JQ973686	KJ174937	KF735645	KC683527	EPI553291	JF965167
27	AY585364	KF042284	JQ973678	KJ719459	KF813115	KC261473	EPI553299	JF965145
28	AY585366	KM027999	JX534565	KP000007	GU477542	KC261474	EPI749123	CY109274
29	AY585369	KP288324	KC261464	KP000015	KJ003989	KC261475	EPI594521	CY109322
30	AY585370	KP762509	KC261465	KP000023	KF042286	KC261476	EF124310	CY109330
31	AY585371	KP762501	KC261466	KJ683880	KM028001	KC261477	EF124218	CY109354
32	AY585372	KU042747	CY091627	KU143255	KP288318	KP233706	EU243127	CY109362
33	AY585373	KU042742	CY091643	KX160201	KP762510	KT587288	DQ997157	CY109370
34	AY585374	KT762439	KM504101	KX160193	KP762502	MK744009	AY741218	CY109410
35	AY585375	KT762431	KP233704	KX160186	KU042805	KJ907633	DQ997077	CY109442
36	AY585376	KU143260	KM251466	KX160179	KU042804	EPI352223	GU182224	CY109562
37	AY585377	KU143259	KM251470	KX160164	KU042802	EPI420387	HM172198	CY109586
38	AY609312	KU143257	KP284957	KX160156	KU042800	EPI279948	HM172207	CY109602
39	AY651348	KP715064	KP284965	MF116313	KT762441	EPI280135	EU243134	CY109626
40	AY651349	KP793723	KP284973	KY437791	KT762433	EF124258	GU182232	CY109642
41	AY651352	KU057296	KP284981	KY437799	KU143342	EF124224	HM172171	CY109674
42	AY651353	KU057280	KP285005	HQ677023	KU143340	EF124225	HM172184	CY109706
43	AY651354	KU057264	KP285013	EPI553223	KP715066	EF124226	HM172204	CY109714
44	AY651358	KX867865	KP285029	EPI553292	KU057282	EF124306	KF638581	CY109722
45	AY651362	KX867873	KP286085	EPI593013	KU057266	EF124303		CY109730
46	AY651366	KY415617	KP286093	EPI594523	KX867867	EF124251		CY109738
47	AY737289	KY415618	KP286101	EPI656794	KX867875	EF124227		CY109746
48	AY737304	MF116309	KP286421	EPI659567	KY415706	CY098580		CY109770
49	AY741213	MF116311	KT313418	EPI750017	MF116319	CY098592		CY109810
50	AY741215	MF116310	KT587286	DQ992794	MF116321	CY098598		CY110178
51	AY741217	KY437767	KT383478	DQ992805	MF116320	EF124190		CY110250
52	AY741221	MK743999	KT963061	EU243126	KY437769	CY030899		CY110276
53	AY950231	MK744015	KU042765	DQ997156	KR010413	CY030923		CY110318
54	CY028924	MF598579	KU042763	AY741217	MK744017	CY030939		CY110406
55	CY028925	KJ907599	KT221082	DQ997076	MK744033	EF124274		CY110422
56	CY028926	KJ907615	KU143269	EU430511	MF598581	EF124239		CY110638
57	CY028927	KJ907639	KP732628	GU182238	KJ907601	EF124248		CY110706
58	CY028929	EPI375432	KP732629	HM172103	KJ907617	EF124269		JX304754
59	CY028933	EPI553219	KP732631	EPI553276	KJ907641	EF124229		JX297583
60	CY028940	EPI553300	KP732634	EPI645959	EPI347305	EF124235		CY146620
61	CY028941	EPI556920	KP732635	GU182222	EPI375433	EF124255		JX304746
62	CY028944	EPI559789	KP732638	HM172094	EPI454494	EF124273		JX304770

63	CY028945	EPI592914	KP732639	HM172111	EPI553217	CY098605		KJ200933
64	CY028953	EPI656522	KP732642	KF638579	EPI553315	CY098650		KJ200917
65	CY028961	EPI656698	KP732643	MF116312	EPI553323	CY098670		KJ200909
66	CY028962	EPI658567	JX094831	EPI553284	EPI556919	FJ784763		KJ200901
67	CY028963	EPI659583	KX523694	EPI659551	EPI749130	CY036055		KJ200893
68	CY028967	EPI659607	KP083451		EPI656521	CY036127		KJ200885
69	CY028968	EPI659663	KT454939		EPI658566	CY036143		KJ200877
70	CY028971	EPI661471	KY415631		EPI658894	GU220788		KJ200869
71	CY028974	EPI661639	KY415646		EPI659582	FJ784762		KJ200861
72	CY028979	EPI661655	KX223685		EPI659606	FJ784773		KJ200853
73	CY030897	EPI665389	MF407528		EPI659662	CY098683		KJ200845
74	CY030929	EPI669768	MF407529		EPI660262	GQ202209		KJ200837
75	CY030985	EPI669784	MF960001		EPI660438	CY098690		KJ200821
76	CY036053	EPI749145	KJ907647		EPI661638	CY098697		KJ200813
77	CY036093	EPI749273	KY005855		EPI661654	CY098704		KJ200789
78	CY036109	EPI749313	KY005871		EPI666500	CY098711		KJ200781
79	CY036133	EPI749329	KY437783		EPI669823	AB521160		KJ200765
80	CY036173	EPI749353	MK744007		EPI749176	CY098718		KJ200757
81	CY036221	EPI749361	MH685179		EPI749216	CY098768		KJ200749
82	CY036245	EPI749393	KY320443		EPI749232	CY098739		KJ200741
83	CY036269	EPI749449	EPI352222		EPI749296	CY098840		KJ200725
84	CY063318	EPI957907	EPI420386		EPI749320	CY098847		KJ200717
85	CY091627	EPI1055344	EPI431448		EPI749360	CY098753		KJ200709
86	CY091643	MN056891	EPI431456		EPI749392	EF670480		KJ200701
87	CY098668	MN165553	EPI530063		EPI749400	DQ095669		KJ200685
88	CY098695	DQ992715	EPI543002		EPI749432	DQ520853		KJ200677
89	CY098702	DQ320911	EPI543010		EPI749440	FJ784772		KJ200669
90	CY098723	DQ320902	EPI557124		EPI749448	FJ784767		KJ200645
91	CY098744	DQ992719	EPI587534		EPI1055322	HM172175		KP285309
92	CY098751	DQ320896	EPI593036		EPI1055327	HM172169		KT267022
93	CY098758	DQ992811	EPI643147		EF124286	HM172195		KT369978
94	CY098766	FJ517645	EPI646024		DQ321031	HM172167		KT369980
95	CY098798	CY036045	EPI656602		DQ321034	CY098824		KT369983
96	CY098822	DQ992741	EPI656650		DQ321044	JX094832		KT369984
97	CY098830	DQ992782	EPI657570		EF124280	EF124304		KT423147
98	CY098845	CY036205	EPI657586		EF124294	EF124219		KU762359
99	CY098853	CY036173	EPI657834		EF124296	CY098586		KU050779
100	CY146644	CY036165	EPI658503		DQ321029	EF124338		KU050772
101	CY146692	CY036093	EPI658911		FJ517646	EF124272		KY415638
102	CY146708	GQ227380	EPI659167		CY036047	EF124203		KY056289
103	DQ095625	AB557629	EPI659559		EF124212	EF124234		KT266926
104	DQ095626	AB521163	EPI659655		CY036207	EF124240		KT267030
105	DQ100556	CY098853	EPI661343		CY036223	EF124253		MG198928
106	DQ201829	CY098744	EPI661903		CY036191	EF124246		MG198925
107	DQ211922	KJ522766	EPI664253		CY036095	CY098643		MG198927
108	DQ211923	HQ636461	EPI664573		GQ227389	CY098657		KJ781227
109	DQ211924	KF572434	EPI671669		AB517662	FJ784766		MH592421
110	DQ320875	KX247927	EPI671693		AB521164	CY036159		HM050392
111	DQ320880	KX960156	EPI671701		AB517664	CY036111		CY110973
112	DQ320881	MF620119	EPI680598		CY098855	FJ784769		MH592254
113	DQ320885	AY651363	EPI684565		CY098746	CY098725		EPI656706
114	DQ320894	AY651366	EPI749417		KJ522767	EF124340		EPI657730
115	DQ320896	AY651365	EPI749505		KF572435	HM172182		EPI658167
116	DQ320902	CY028961	EPI749561		KX247922	HM172166		EPI658215
117	DQ320911	DQ320894	EPI749625		KX247928	CY098832		EPI659343
118	DQ320927	DQ320895	EPI749737		KX247937	EF124220		EPI659527
119	DQ343150	EU635874	EPI759966		KX960151	EF124250		EPI660935
120	DQ366338	DQ095626	EPI873669		KX960163	EU128239		EPI661743
121	DQ371928	DQ320925	EPI958800		MF620124	CY030987		EPI665309
122	DQ371930	DQ320898	EPI959050		MH127651	EF124200		EPI666565
123	DQ432037	DQ992751	EPI961183		AY651475	EF124245		EPI666589
124	DQ432045	CY036197	EPI1009536		CY029260	EF124332		EPI667109
125	DQ659327	GU182150	EPI1055368		CY029316	EF124231		EPI667285
126	DQ822563	JQ638674	EPI1060731		CY029323	EF124236		EPI671589
127	DQ914814	JX534557	EPI1060753		DQ321026	CY098617		EPI671637
128	DQ992719	KF813113	EPI1091684		DQ321027	CY098784		EPI687493

129	DQ992741	GU477549	EPI1093310		DQ321055	EF124238		EPI717861
130	DQ992747	KP288316	EPI1202729		EU635875			EPI717869
131	DQ992755	KU057288	EPI1258049		EF124283			EPI717877
132	DQ992756	KJ907607	EPI1326815		DQ095667			EPI717893
133	DQ992765	EPI656530	EPI1352813		DQ520854			EPI717909
134	DQ992778	EPI665317	EPI1352829		DQ321057			EPI717917
135	DQ992790	EPI665709	EPI1369965		EF124210			EPI717933
136	DQ992833	EPI669792	EPI1394202		CY036263			EPI717941
137	DQ992842	EPI749281	EPI1394708		GU182200			EPI717949
138	DQ992844	EPI749369	EPI1426910		HM172210			EPI717973
139	DQ997087	EPI749385	EPI1435113		JN646728			EPI749921
140	DQ997094	DQ992723	EPI1435209		JQ638677			EPI749969
141	DQ997122	DQ992726	FJ492880		KP288326			EPI749977
142	DQ997147	DQ992812	FJ492883		KU057290			MH592247
143	DQ997156	CY036181	FJ492884		KY415705			MH592248
144	DQ997172	GQ227383	MN173395		KY776497			MH592250
145	DQ997182	AB517661	DQ992735		MK744001			MH592253
146	DQ997308	AB517663	DQ992754		EPI656713			MH592256
147	DQ997318	KX247915	DQ992756		EPI660638			MH592257
148	DQ997355	MH127649	DQ992787		EPI669855			MH592258
149	DQ997392	DQ320877	DQ992729		EPI749144			MH592259
150	DQ997405	CY028970	DQ992738		EPI749288			MH592260
151	DQ997410	DQ320923	DQ371928		EPI749344			MH592261
152	DQ997531	CY036237	DQ371930		EPI749352			MH592263
153	DQ997547	GU182198	FJ492885		EPI749456			MH592264
154	EF587277	KU042746	FJ492882		EPI1055316			MH592265
155	EF670482	KU143258	DQ842487		EPI1055319			MH592266
156	EPI1055368	EPI347304	DQ992842		DQ321037			MH592267
157	EPI1060731	EPI553308	CY030945		DQ321028			MH592268
158	EPI1093310	EPI560152	CY030897		EF124292			MH592275
159	EPI1144449	EPI660639	DQ992836		EF124209			MH592277
160	EPI1145868	EPI666501	DQ992786		GQ227381			CY109951
161	EPI1202729	EPI669776	DQ992844		AB521162			CY110255
162	EPI1258057	EPI749201	DQ992838		KX960143			CY109957
163	EPI1326815	EPI749225	DQ993030		KX960147			CY109995
164	EPI1333877	EPI749377	DQ992790		KX960157			CY110071
165	EPI1333923	EPI749401	DQ993117		DQ321009			CY110075
166	EPI1352813	EPI1055323	DQ992783		AY651478			CY110039
167	EPI1352861	EPI1055328	FJ492881		DQ321056			CY110475
168	EPI1394202	DQ320905	FJ492879		CY036199			CY110661
169	EPI1394708	DQ320912	CY098634		CY036255			CY110099
170	EPI1426910	DQ992797	CY098641		GU182152			CY110677
171	EPI1435113	DQ992716	CY098648		HM172178			CY110149
172	EPI347304	CY036189	FJ784843		GU477543			CY110555
173	EPI352222	GQ227391	FJ784846		KU057298			HQ599858
174	EPI375432	AB557633	CY036053		KX571058			HM800947
175	EPI420386	AB521161	CY036125		KJ907609			KT343660
176	EPI431448	KX247933	CY036133		EPI553307			KT343653
177	EPI431456	KX960142	GU220793		EPI559729			KP767407
178	EPI530063		FJ784842		EPI661470			KP767410
179	EPI543010		CY098681		EPI669783			KP767453
180	EPI553219		GQ202211		EPI749184			KT343650
181	EPI553292		CY098688		EPI749376			MH352276
182	EPI553308		CY098695		EF124282			MH352277
183	EPI592914		CY098702		EF124287			HM144520
184	EPI594523		CY098709		DQ321043			HM144501
185	EPI645967		AB521159		EF124213			HM144502
186	EPI646024		CY098716		CY036167			HM144475
187	EPI656602		CY098723		AB557630			JF965162
188	EPI656618		CY098730		KX247916			JF965143
189	EPI656842		CY098845		AY651476			JF965154
190	EPI657562		CY098751					JF965138
191	EPI657586		JQ973670					CY110949
192	EPI657834		JQ973694					CY109394
193	EPI658919		KT936709					CY109554
194	EPI659175		MG220416					CY109610

195	EPI659559		MK027087					CY109634
196	EPI659567		EF670479					CY109690
197	EPI659575		DQ095629					CY110682
198	EPI659655		DQ520855					KJ200653
199	EPI659663		DQ992830					KT369979
200	EPI660431		DQ992755					KU050780
201	EPI660727		DQ992762					KT266990
202	EPI661655		FJ784855					MG198926
203	EPI662031		EU195392					KJ781219
204	EPI668767		GU182214					EPI556912
205	EPI671701		HM172093					EPI656586
206	EPI687156		HM172107					EPI658271
207	EPI749313		HM172104					EPI658559
208	EPI749329		HM172109					EPI658607
209	EPI749369		KC683523					EPI658679
210	EPI749865		KC261463					EPI659647
211	EPI759966		KC261467					EPI660815
212	EPI961183		KP285021					EPI661871
213	EU195400		KT370062					EPI666797
214	EU195416		KU143270					EPI667069
215	EU434686		KP732641					EPI667101
216	EU635874		KP732646					EPI717901
217	FJ492883		KU852944					EPI749929
218	FJ492884		KU057272					EPI749945
219	FJ517645		KX013021					EPI749993
220	FJ602794		KT952272					EPI752973
221	FJ602799		KY415622					MH592251
222	FJ602807		KY415633					CY109945
223	FJ602866		KY056285					CY109947
224	FJ784842		KY056286					CY110053
225	FJ784851		KY437807					CY109961
226	FJ784852		MF399660					CY109983
227	GQ184330		KY393304					CY109931
228	GQ227380		MH156492					CY110363
229	GU052019		KJ907631					CY110385
230	GU052057		EPI557108					CY110011
231	GU052065		EPI645967					CY110015
232	GU052089		EPI656642					CY110081
233	GU052465		EPI656722					CY110083
234	GU182166		EPI658751					CY110017
235	GU182190		EPI659047					CY110025
236	GU182238		EPI659175					CY110671
237	GU186692		EPI659919					CY110111
238	GU220793		EPI661631					CY110567
239	GU477549		EPI661663					KP767528
240	GU596984		EPI661839					MH352273
241	GU727653		EPI665981					HM144486
242	GU727661		EPI666077					HM144527
243	GU727677		EPI671605					HM144514
244	HM172069		EPI687523					HM144530
245	HM172073		EPI749337					HM144504
246	HM172075		EPI749521					HM144494
247	HM172076		EPI749649					HM144500
248	HM172077		EPI956143					JF965153
249	HM172078		EPI1208376					JF965155
250	HM172079		EPI1055332					CY110941
251	HM172080		EPI1144246					CY109482
252	HM172081		EPI1144449					CY109506
253	HM172086		EPI1258041					CY109666
254	HM172087		EPI1333902					CY109754
255	HM172089		EPI1333910					CY109802
256	HM172090		EPI1333923					CY109866
257	HM172092		EPI1352861					CY109914
258	HM172095		EPI1574313					CY110446
259	HM172096		DQ992757					CY110524
260	HM172098		DQ992777					CY110622

261	HM172100		CY030985					CY110646
262	HM172101		DQ992763					JX304762
263	HM172105		DQ992841					KJ200925
264	HM172113		DQ992839					KJ200733
265	HM172114		DQ992833					KJ200661
266	HM172116		DQ992793					KT369985
267	HM172454		DQ992784					KT423143
268	HM172455		CY036141					KU143274
269	HQ636461		CY036157					KU050769
270	HQ677023		CY098838					KY415615
271	JF732739		KX247930					KT266998
272	JF975561		MF362113					MH592413
273	JN646713		DQ992822					EPI658199
274	JN646714		EU594349					EPI658511
275	JQ041401		HM172072					EPI659071
276	JQ277225		HM172112					EPI660823
277	JQ638673		CY098830					EPI665653
278	JQ973670		KJ938658					EPI717957
279	JQ973678		KM251464					EPI749937
280	JQ973686		KT221066					CY110155
281	JQ973694		KU143271					CY109955
282	JX507355		KP732644					CY109967
283	JX534549		KR063687					CY109971
284	JX534565		KY415619					CY110365
285	JX534573		KY415623					CY110087
286	JX534581		KY415644					CY109919
287	JX534589		KX602690					CY109927
288	JX565019		MF960000					KP767452
289	JX576788		MF399572					JF965166
290	KC261464		EPI656778					CY109418
291	KC261466		EPI660207					CY109522
292	KC261467		EPI660351					CY109538
293	KC357320		EPI660975					CY109578
294	KC683522		EPI661039					CY110170
295	KF042284		EPI661567					CY110334
296	KF572434		EPI664381					KU050775
297	KF638579		EPI671629					KU050774
298	KJ003987		EPI671677					KU572418
299	KJ522766		EPI684541					EPI659503
300	KJ719459		EPI687156					EPI660951
301	KJ907599		EPI749473					EPI660967
302	KJ907631		EPI749681					EPI665725
303	KJ907647		EPI749777					EPI666877
304	KJ933377		EPI858828					EPI667061
305	KM027999		EPI1215862					EPI667085
306	KM251466		EPI1009528					EPI671565
307	KM251470		EPI1055335					EPI717981
308	KP233704		EPI1333877					EPI749913
309	KP284957		EPI1333894					CY109939
310	KP284965		MN173482					CY109953
311	KP284981		MN173506					CY109959
312	KP285013		CY030969					CY109989
313	KP285021		DQ992742					CY110387
314	KP286085		CY098655					CY109378
315	KP286093		CY098668					CY109474
316	KP732628		FJ784844					CY110268
317	KP732629		FJ784845					KJ200773
318	KP732631		CY036061					KJ200693
319	KP732634		CY036077					KT423148
320	KP732635		FJ784853					EPI656554
321	KP732643		CY098766					EPI658423
322	KP762501		KT936708					EPI658439
323	KT221066		KM392379					EPI659399
324	KT313418							EPI660247
325	KT370062							EPI661159
326	KT587286							EPI666125

327	KT762431							EPI666701
328	KT762439							EPI749905
329	KT936695							EPI749961
330	KT963061							CY109941
331	KU042747							CY109973
332	KU042765							CY109997
333	KU042768							CY110073
334	KU057264							CY110037
335	KU057280							CY110041
336	KU143255							CY110089
337	KU143269							JF965146
338	KX094408							CY109402
339	KX160148							CY109570
340	KX160186							CY110614
341	KX223685							KJ200797
342	KX867865							KU050771
343	KX960178							KU572410
344	KX960184							KT266918
345	KY005863							EPI660903
346	KY056285							EPI661695
347	KY415617							EPI661711
348	KY415618							MH592271
349	KY415619							CY109935
350	KY415631							CY110033
351	KY415633							CY110051
352	KY437799							KT343652
353	KY776496							
354	MF040672							
355	MF116309							
356	MF116310							
357	MF116311							
358	MF116312							
359	MF182415							
360	MF620120							
361	MF960000							
362	MF960001							
363	MG220416							
364	MK027087							
365	MK743999							
366	MK744007							
367	MK744015							
368	MN056891							
369	MN165553							
